# Sciadonic acid derived from pine nuts as a food component to reduce plasma triglycerides by inhibiting the rat hepatic Δ9-desaturase

**DOI:** 10.1038/s41598-020-63301-3

**Published:** 2020-04-10

**Authors:** Frédérique Pédrono, Nathalie Boulier-Monthéan, Françoise Boissel, Jordane Ossemond, Roselyne Viel, Alain Fautrel, Justine Marchix, Didier Dupont

**Affiliations:** 1 AGROCAMPUS OUEST, Rennes, France; 2 INRAE Science et Technologie du Lait et de l’Œuf, équipe Bioactivité et Nutrition, Rennes, France; 3 INRAE Science et Technologie du Lait et de l’Œuf, équipe Séchage, Matrices concentrées et Fonctionnalités, Rennes, France; 4 Université de Rennes1, Inserm, CNRS, Plateforme d’histopathologie H2P2, Biosit, Biogenouest, Rennes, France; 5 Cincinnati Children’s Hospital Medical Center, Division of Pediatric General and Thoracic Surgery, Cincinnati, United States

**Keywords:** Lipids, Metabolic disorders

## Abstract

Sciadonic acid (Scia) is a Δ5-olefinic fatty acid that is particularly abundant in edible pine seeds and that exhibits an unusual polymethylene-interrupted structure. Earlier studies suggested that Scia inhibited the *in vitro* expression and activity of the Stearoyl-CoA Desaturase 1 (SCD1), the hepatic Δ9-desaturase involved in the formation of mono-unsaturated fatty acids. To confirm this hypothesis, rats were given 10% Scia in diets balanced out with n-6 and n-3 fatty acids. In those animals receiving the Scia supplement, monoene synthesis in the liver was reduced, which was partly attributed to the inhibition of SCD1 expression. As a consequence, the presence of Scia induced a 50% decrease in triglycerides in blood plasma due to a reduced level of VLDL-secreted triglycerides from the liver. In non-fasting conditions, results showed that Scia-induced inhibition of SCD1 led to a decrease in the proportions of 16:1n-7 and 18:1n-7 in the liver without impacting on the level of 18:1n-9, suggesting that only triglycerides with neosynthesized monoenes are marked out for release. In conclusion, this *in vivo* study confirms that Scia highly inhibits SCD1 expression and activity. The work was performed on normo-triglyceride rats over six weeks, suggesting promising effects on hyper-triglyceridemic models.

## Introduction

Sciadonic acid (Scia) is composed of an aliphatic chain with 20 carbons and 3 unsaturations (20:3Δ5,11,14). It is a Δ5-olefinic acid, a fatty acid (FA) group characterized by the presence of a polymethylene-interrupted double bond at C5, as distinct from the more common malonic structure. In the natural environment, Scia is specifically abundant in conifers, although it is also present in trace amounts with different other olefinic structures in slime molds^1^ and certain marine invertebrates^2^.

Seven Δ5-olefinic acids are commonly found in gymnosperms and their composition profile provides a good marker of the plant phylogeny^3,4^. Scia is particularly associated with the suborder Taxares, representing up to 26% of the total FA content in *Podocarpus nagi*^5^. It is ubiquitously found in pine nuts, constituting up to 7% of the total FA in *Pinus pinaster*^6^. As it is present in edible seeds and consumed as a nutrient, studies performed on rodent models have been carried out to determine its functional effect. Scia is incorporated in membranes when supplied to the cells and substitutes for arachidonic acid (20:4n-6) in phospholipids such as phosphatidylinositol^7^. Consequently, this unusual FA integrates lipid metabolism resulting in the modulation of physiological functions. Scia supplemented to mammal cells induces synthesis of the uncommon 16:2n-6 (Δ7,10), which is subsequently elongated into linoleic acid (18:2n-6 or Δ9,12)^8,9^. Scia then reduces the production of modulators causing inflammation that are derived from arachidonic acid^10–13^. In rats fed with a Scia-enriched diet, triglyceride levels are lowered in the blood plasma and the liver^14–16^.

Previous work performed *in vitro* on hepatocytes showed that Scia inhibited the expression and activity of the Δ9-desaturase called Stearoyl-CoA Desaturase 1 (SCD1)^17^. This enzyme introduces a *cis* double bound at C9 of saturated FA as acyl-CoA substrate to produce monoenes. Consequently, Scia induced a reduction in such monoenes as 16:1n-7 and 18:1n-9, leading ultimately to the decrease in the secretion of neosynthesized triglycerides. Starting with this hypothesis, the inhibition of SCD1 was investigated *in vivo* on rats fed with a diet enriched in Scia. The specificity of the present study rests on the control diet which was supplemented by using medium chain FA (10:0 and 12:0) to compensate for the Scia used in the test diet. This choice was motivated by the necessity of maintaining the content of C16 and C18 FA provided by the diet, with the aim of underlining the specific impact of Scia on the FA metabolism induced by SCD1.

## Materials and methods

### Animals, diets and tissue sampling

All handling protocols performed complied with the European Union Guideline for animal care and use (2010/63/CEE; decree 2013-118). The present project was evaluated by the regional ethic committee (Comité Rennais d’Ethique en matière d’Expérimentation Animale) and was authorised by the French Ministry of Higher Education and Research under the number 3882-2016111717375510-v1. Male Sprague-Dawley rats (3 weeks-old) were supplied by the Janvier Labs Breeding Center (Le Genest-Saint-Isle, France). The provided litter was made from pine shaving (Special Diet Services, Augy, France), that contained Scia (242 µg/g). Animals had access to water and food *ad libitum* in controlled conditions to avoid food oxidation^18^. They were fed for 6 weeks with either a control diet or the Scia diet, both composed of 22.2% proteins, 60.3% carbohydrates, 2.0% fibers, 5.5% mineral and vitamin mix^19^ of the dry diet (Unité de Préparation des Aliments Expérimentaux of INRA, Jouy en Josas, France). Pellets were then formulated with 10% of lipids by adding an oil mixture. In the case of the control diet, the mix contained 25% olive oil, 45% walnut oil, 10% corn oil, 10% capric ethyl ester and 10% lauric ethyl ester. In the case of the Scia test diet, the oil mix contained 25% olive oil, 45% walnut oil and 30% purified pine oil (Polaris, Quimper, France) composed of ethyl esters, as opposed to the other vegetable oils composed of triglycerides.

Non-fasted rats were anesthetized with intraperitoneal injections of ketamine (100 mg/kg, Imalgene®1000, Mérial, Lyon, France) and xylazine (10 mg/kg, Rompun® 2%, Bayer Animal Health, Puteaux, France). Blood was collected in heparin tubes by cardiac puncture. Plasma was separated from red blood cells (RBC) by centrifugation (2000rpm, 15 min, 15 °C). Fractions of plasma such as very low density lipoproteins (VLDL) were obtained on OptiPrep™ density gradient medium^20^ as follows: 1.5 mL of plasma was mixed with 12% iodixanol and then centrifuged at 278 000 g for 4 hours and 20 min at 16 °C with a slow deceleration. Fifteen fractions of 180 µL each were finally collected. Liver, brain, heart, lungs, testis and subcutaneous white adipose tissue (WAT) were all sampled, snap-frozen and then stored at −80 °C until analysis. Those liver samples to be used for qPCR analysis were first grounded in Trizol® reagent (Life Technologies, Saint-Aubin, France).

### Lipid analyses

Lipids were extracted using Folch’s method^21^ and quantified after weight measurement. The FA profile was determined from FA methyl esters (FAME) as described previously^17^. In this procedure, lipids were saponified and FA were methylated to be analyzed on an Agilent gas chromatograph (7890) equipped with a BPX70 capillary column and coupled to a flame ionization detector (Agilent Technologies, Les Ulis, France). The identification of FA was checked by mass spectrometry on a QP 2010-SE gas chromatograph (Shimadzu, Marne-La-Vallée, France) by using the NIST mass spectral database library (2017). 4,4-Dimethyloxazoline (DMOX) derivatives were also prepared on lipids from the diet and tissues to confirm the presence of unusual FA like 20:2Δ5,11 and 22:3Δ7,13,16. Results were expressed as a percentage of the total FA and concentrations were calculated by using 17:0 as the internal standard. The relative results of the effect of the Scia diet are mainly presented as the % of the total FA excluding the Scia: thus, percentages were recalculated without considering the GC area of Scia. In this way, the incorporation of Scia was distinguished from its effect on FA metabolism. Noting that the concentration of Scia was negligible in tissues (less than 0.25%, Table 1), it was deduced that its incorporation into the tissue led to a reduction of the other FA proportions.

**Table 1:** The profile of Scia and monoenes in tissue samples.

		Total FA	Scia		16:1n-7		18:1n-7		18:1n-9	
		µg/mg or µL	%	ng/mg or µL	% ^‡^	µg/mg or µL	% ^‡^	µg/mg or µL	% ^‡^	µg/mg or µL
**Plasma**	**Control**	**5.0** ± **0.2**	**0.24** ± **0.01**	**12.2** ± **0.6**	**2.3** ± **0.2**	**0.12** ± **0.02**	**2.7** ± **0.2**	**0.13** ± **0.01**	**18.3** ± **0.5**	**0.92** ± **0.06**
	Scia	3.5 ± 0.2*	5.76 ± 0.16*	200.8 ± 12.2*	1.1 ± 0.1*	0.04 ± 0.01*	1.6 ± 0.1*	0.05 ± 0.06*	14.3 ± 0.5*	0.47 ± 0.03*
**Liver**	**Control**	**38.1** ± **1.7**	**0.18** ± **0.01**	**69.7** ± **4.0**	**2.9** ± **0.4**	**1.15** ± **0.20**	**4.1** ± **0.2**	**1.58** ± **0.15**	**19.2** ± **0.7**	**7.36** ± **0.54**
	Scia	33.9 ± 2.1	3.37 ± 0.17*	1145.4 ± 94.3*	1.8 ± 0.2*	0.60 ± 0.09*	2.8 ± 0.2*	0.92 ± 0.06*	19.8 ± 1.1	6.62 ± 0.77
**RBC**	**Control**	**2.3** ± **0.1**	**0.08** ± **0.02**	**1.8** ± **0.5**	**0.4** ± **0.0**	**0.01** ± **0.00**	**2.8** ± **0.1**	**0.07** ± **0.00**	**8.0** ± **0.3**	**0.19** ± **0.01**
	Scia	2.2 ± 0.1	3.46 ± 0.19*	76.0 ± 7.6*	0.2 ± 0.0*	0.00 ± 0.00*	2.0 ± 0.1*	0.04 ± 0.00*	6.9 ± 0.2*	0.15 ± 0.01*
**Brain**	**Control**	**22.7** ± **1.4**	**0.00** ± **0.00**	**0.0** ± **0.0**	**0.4** ± **0.0**	**0.09** ± **0.01**	**2.9** ± **0.1**	**0.66** ± **0.06**	**13.8** ± **0.4**	**3.16** ± **0.29**
	Scia	26.7 ± 1.0*	0.35 ± 0.01*	93.7 ± 7.0*	0.4 ± 0.0	0.10 ± 0.01	2.8 ± 0.1	0.76 ± 0.05	14.4 ± 0.3	3.84 ± 0.21
**Heart**	**Control**	**18.8** ± **0.5**	**0.02** ± **0.01**	**4.8** ± **2.2**	**0.3** ± **0.0**	**0.06** ± **0.01**	**3.3** ± **0.1**	**0.62** ± **0.02**	**6.1** ± **0.4**	**1.15** ± **0.09**
	Scia	18.9 ± 0.9	2.44 ± 0.08*	461.4 ± 24.4*	0.2 ± 0.0*	0.03 ± 0.00*	2.6 ± 0.1*	0.49 ± 0.03*	5.2 ± 0.2*	0.95 ± 0.05
**Lung**	**Control**	**18.7** ± **0.9**	**0.20** ± **0.02**	**36.8** ± **4.2**	**1.5** ± **0.1**	**0.30** ± **0.04**	**1.9** ± **0.2**	**0.35** ± **0.03**	**14.6** ± **1.4**	**2.80** ± **0.42**
	Scia	19.6 ± 1.7	2.27 ± 0.02*	444.1 ± 34.4*	1.3 ± 0.2	0.27 ± 0.05	1.4 ± 0.1*	0.27 ± 0.03	16.6 ± 1.3	3.33 ± 0.58
**WAT**	**Control**	**297.2** ± **27.9**	**0.02** ± **0.00**	**60.5** ± **14.8**	**3.9** ± **0.1**	**11.76** ± **1.20**	**2.4** ± **0.1**	**7.33** ± **0.88**	**32.8** ± **0.3**	**97.63** ± **9.93**
	Scia	322.1 ± 36.8	2.22 ± 0.08*	7139.0 ± 823.6*	3.6 ± 0.4	12.27 ± 2.65	1.9 ± 0.1*	5.92 ± 0.83	31.7 ± 0.2*	99.72 ± 11.10
**Testis**	**Control**	**11.3** ± **0.9**	**0.12** ± **0.01**	**13.2** ± **1.7**	**1.3** ± **0.3**	**0.16** ± **0.04**	**2.3** ± **0.1**	**0.26** ± **0.03**	**13.3** ± **1.4**	**1.60** ± **0.29**
	Scia	11.2 ± 1.7	1.51 ± 0.16*	168.0 ± 55.4*	1.2 ± 0.4	0.18 ± 0.10	2.2 ± 0.1	0.25 ± 0.04	13.0 ± 1.8	1.63 ± 0.57

Lipids were extracted from tissues by Folch’s method and the FA profile was determined by GC. The total FA were quantified as reported for each diet. The level of incorporation of Scia and the proportion of monoenes are also presented. Results are presented in percentage of total FA (^‡^excluding Scia) or as concentrations expressed per mg of tissues or µL of plasma. The significance of the difference between diets was estimated by a *t*-test (*p < 0.05).

Lipids from plasma and liver were separated on silica thin layer chromatography (TLC) using the mixture hexane: diethyl ether: acetic acid (70:30:1, v/v). The bands of lipids were observable by spraying the plates with a 10% (w/v) cupric sulfate solution in 8% (w/v) orthophosphoric acid and heating for 20 min at 150 °C. The density of bands was estimated by using Image J software (US National Institute of Health, www.nih.gov). On a second plate, lipids were observable by spraying the plate with a 5% (w/v) primuline solution in a mixture of acetone: water 80:20 (v/v). The bands corresponding to triglycerides and phospholipids were scrapped off and analyzed as described above to determine their FA profile. Triglycerides and cholesterol were separately quantified in plasma by using a enzymatic kit from DiaSys (Grabels, France). Oxidized derivatives of FA (18:2n-6, 20:4n-6, 20:5n-3 and 22:6n-3) were quantified by LC-QQQ Agilent 6460 (Justine Bertrand-Michel and Pauline Le Faouder, MetaToul-Lipidomique Core Facility, I2MC, Inserm 1048, MetaboHUB-ANR-11-INBS-0010 Toulouse, France)^22^.

### Hepatic mRNA relative expression

Total RNA was extracted with Trizol® (Life Technologies, Saint-Aubin, France) and reverse transcribed by using the high capacity cDNA RT kit (Applied, Fisher, Illkirch, France). Real-time PCR of genes encoding for the desaturases (Taqman®) and for the other proteins (SYBR®Green) was carried out as described in previous works^17,23^. The mRNA expression was evaluated as a delta Cycle threshold with 18S as reference for Taqman® and glyceraldehyde 3-phosphate dehydrogenase (GAPDH) as reference for SYBR®Green: ΔCt = Ct_gene_ - Ct_reference_. Results obtained from triplicates per animal were expressed from the 2^-ΔCt^ calculation.

### Protein expression in the liver

The expression of Δ9-desaturase and SREBP-1c (Sterol Regulatory Element Binding Protein-1c) was estimated by western-blot after SDS-PAGE. Goat anti-SCD1 (Santa-Cruz Biotechnology, Heidelberg, France), mouse anti-SREBP1 (Abcam, Paris, France) and mouse anti-GAPDH (Sigma-Aldrich, Saint-Quentin Fallavier, France) were all used following the manufacturer instructions. Primary antibodies were coupled to horseradish peroxidase-conjugated anti-IgG and the peroxidase activity was determined by chemiluminescent detection using Immobilon reagents (Millipore, Molsheim, France). The density of bands was estimated by using Image J software.

### Liver immunostaining

The immunohistochemical staining was performed on samples of the liver that had been fixed by formalin and embedded on paraffin. Histological measurements were completed on 4 µm sections cut on a Leica RM 2145 microtome at the Histopathology Facility H2P2 (Biogeneouest, Université de Rennes I, France). Sections were mounted on positively charged slides and dried at 56 °C for 60 min. Immunohistochemical staining was performed by a Ventana Discovery XT Automated IHC stainer (ROCHE Basel Switzerland) using the Ventana OmniMap detection kit. Following removal of the paraffin (at 75 °C for 8 min) antigen retrieval was done with Tris-EDTA buffer solution CC1 (pH8), at 95 °C to 100 °C for 28 min. Endogenous peroxidase was blocked by treating with Inhibitor-D 3% H_2_O_2_ for 10 min at 37 °C. The slides were incubated at 37 °C for 60 min with mouse anti-SCD1 (5 µg/mL, Abcam) and anti-SREBP1 (2 µg/mL, Abcam). Signal enhancement was performed using the goat anti-mouse horseradish peroxidase (ROCHE®). Slides were then counterstained for 4 min with hematoxylin, rinsed and manually dehydrated. Thereafter, the stained sections were scanned with the NanoZoomer 2.0 RS (Hamamatsu, Tokyo, Japan). The level of brown staining was quantified by use of NIS -Elements software (Nikon).

### Statistics

Results are expressed as the mean ± SEM of 8 animal samples per group. Data analysis was performed using R and Graph Pad software. The significance of the effect of the Scia supplement was evaluated by t-test. The analysis of variance was done punctually by a Sidak’s multiple comparison test as indicated in the legend of the figures. The strength of any correlations found is given by a Pearson coefficient.

## Results

### Scia and other fatty acids in the diets

**Figure 1:**
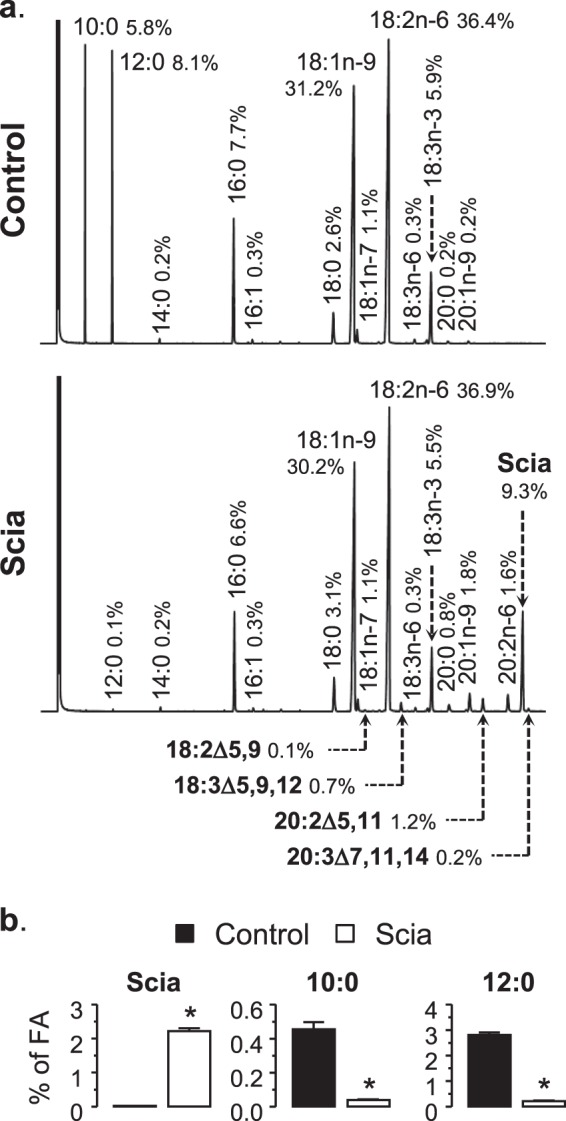
The fatty acid profile of the diets. (**a**) Three-week-old rats were fed for 6 weeks with either the control diet or the test diet (Scia). The FA profile of formulated pellets is given by the chromatograms. Mass percentages of FA are indicated by the side of each peak. (**b**) The specificity of each diet was evaluated at the end of the experiment by profiling the FA of the white adipose tissue. The difference between the two diets was evaluated by a *t*-test (*p < 0.05).

Weanling rats were fed for 6 weeks with either a control diet or a Scia diet. Scia and other Δ5-olefinic acids (and their derivatives) represent respectively 9.3% and 2.2% of total FA (1.95% and 0.48% of energy) in the pellets (Fig. 1a). The purified pine oil used in the trial was predominantly composed of Scia, although the chromatogram showed the presence of very minor Δ5-olefinic acids, such as 20:2Δ5,11 (keteleeronic acid), 18:2Δ5,9 (taxoleic acid) and 18:3Δ5,9,12 (pinolenic acid). The elongation product of the latter, 20:3Δ7,11,14 (bishomo-pinolenic acid), was also present in trace amount. The other FA of the diet, apart from 20:1n-9, were from the vegetable oils chosen to sustain the nutritional equilibrium and to optimize the n-6/n-3 balance. In the control diet, Scia was replaced by the fatty acids 10:0 and 12:0, to maintain a comparable profile for the FA range 16:0 to 18:2n-6. Linoleic acid (18:2n-6) was the most abundant FA in the diets: its proportion (36% of total FA) is intermediate between rat maternal milk (around 25% of total FA) and standard rodent chow (50% of total FA). This formulation choice was made because of its expected increase in tissues following the ingestion of Scia^8,17^ and also to avoid a potential damaging side effect from an excessive level of this FA. Oleic acid (18:1n-9) represented around 30% of the total FA in the diets, which is substantially higher than found in rat milk and chow (about 20% of total FA). The ratio of 18:2n-6 to 18:3n-3 was 6.2 and 6.7 for the control and Scia diets respectively. In these conditions, similar growth of animals and food intake were observed (not shown). The difference between diets was confirmed by analyzing the FA profiles of the white adipose tissue. These results showed that only Scia was accumulated in the tissues of those rats fed with the Scia diet, whereas 10:0 and 12:0 were retained in its place for those fed the control diet (Fig. 1b).

### Metabolism of Scia in rat tissues

Addition of Scia to the diet led to an enrichment of the tissues with this unusual FA, although its presence was also observed in trace amounts in the tissues of animal fed with the control diet (Table 1). Scia was more abundant in blood, and particularly in the plasma, representing 0.24% and 5.76% of total FA for rats fed the control and Scia diets respectively. In the liver samples, Scia represented less than 4% of total FA content with rats fed the Scia diet (1.1 µg per mg of tissue). The brain was an exception as Scia was not detected in the control group. However, the Scia diet induced its accretion to the amount of 94.2 ng per mg of tissue. With respect to the other Δ5-olefinic acids and their derivatives, 20:3Δ7,11,14 was identified in all studied tissues (Supplemental Fig. 1). It was found in trace amounts in tissues (≤0.15%), whereas it was, with its precursor (18:3Δ5,9,12), insignificantly present in the Scia diet (Fig. 1a). In addition, Scia can be elongated into 22:3Δ7,13,16 as previously showed in cultured cells^8,9^ but in contrast with *in vivo* experiments^15,16,24^. In the present trial, this bishomo-sciadonic acid was identified by using DMOX derivatives and GC-MS. Results showed that the component was only detectable in lung (0.41%) and to a lesser extent in white adipose tissue (0.04%) (Supplemental Fig. 1). To summarize, the impact of the Scia diet was evident through the detection of unusual FA, although present at a low level, in the rat tissues.

### Effect of the Scia diet on the FA profiles in rat tissues

We further investigated the effect of Scia on FA content of different tissues. The first observation was that the Scia diet induced a reduction by 30% of the FA content in plasma (Table 1). A similar tendency was observed in liver samples (p = 0.077). On the other hand, the FA concentration in samples of brain tissue was increased by 18% as a consequence of this diet. Concentrations of FA in the other tissues were not significantly affected. The second observation was that the Scia diet induced an overall increase in the n-6 FA proportion, such as 18:2n-6 or its derivatives (Supplemental Fig. 2), in particular in the liver as already reported^8,17^. The FA 16:2n-6 was undetected in the liver tissue whereas it was present in cultured hepatocytes as previously described^17^. Of all the observations, the most remarkable effect was on the monoenes. The Scia diet reduced the 16:1n-7, 18:1n-7 and 18:1n-9 proportions depending on the tissues (Table 1). The most prominent effect was observed on blood plasma with a decrease in proportions of 16:1n-7, 18:1n-7 and 18:1n-9 by respectively 52%, 41% and 22%. The liver was also impacted with a significant decrease in the proportions of 16:1n-7 (−38%) and 18:1n-7 (−32%) but not of 18:1n-9. The concentration of the latter decreased, however, from 7.4 µg/mg with the control diet to 6.6 µg/mg with the Scia diet. Considering the most pronounced effects of the Scia diet, analyses were subsequently focused on plasma and liver samples.

### Effect of the Scia diet on blood plasma

**Figure 2:**
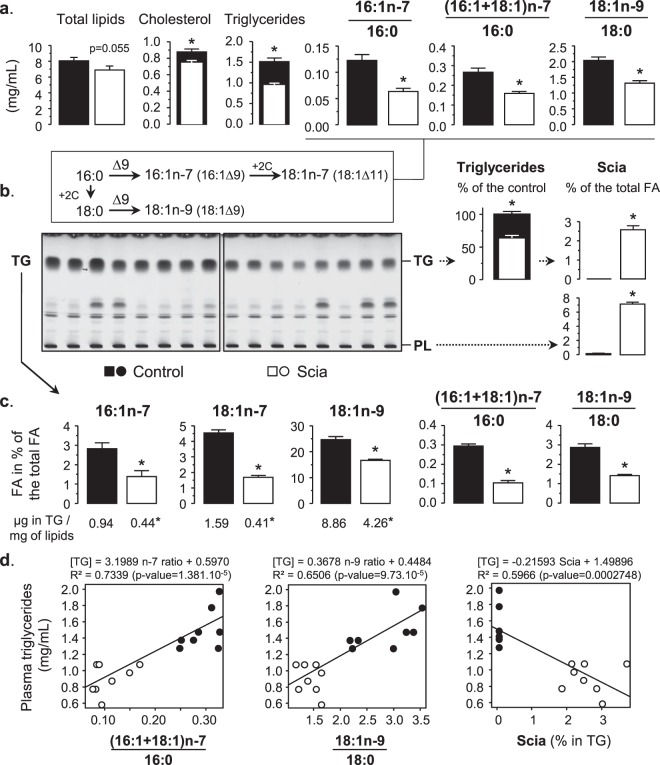
The effect of Scia on the lipid profile of blood plasma. Lipids of blood plasma were extracted by Folch’s method and the FA profile was determined by GC. Lipid classes were separated by TLC and the FA from triglycerides and phospholipids were further analyzed by GC. (**a**) Lipid content and Δ9-desaturase indexes were determined from total lipids. (**b**) The TLC profiles were performed depending on the diets (20 µL of plasma per well). Triglycerides (TG) were estimated by relative quantification of the density of bands. The incorporation of Scia was determined by GC on both triglycerides and phospholipids. (**c**) Monoenes and Δ9-desaturase indexes were determined on the triglyceride fractions. Results on FA from TG are expressed as a % of total FA including Scia. (**d**) The correlation between the triglyceride concentrations in plasma and the n-7/n-9 ratios or Scia percentages of TG was determined for each diet. The significance of the difference between diets was evaluated by a *t*-test (*p < 0.05).

The effect of diets was first analyzed on plasma. The lipid content of plasma from the control diet, including triglycerides and cholesterol, was coherent with a previous work performed on the same rat strain fed with standard rodent chow^25^ (Fig. 2a). This sustains that the choice of medium chain FA was relevant at this point of the trial. Then the Scia diet reduced the lipid content of blood plasma by −14% (p = 0.0547) (Fig. 2a). More specifically, it significantly decreased the cholesterol and triglyceride concentrations by 16% and 38% respectively. These results are concomitant with the reduction of the Δ9-desaturase indexes induced by the Scia diet. The decrease in the synthesis of monoenes by the liver when rats were fed with the Scia diet was confirmed from both the 16:0 and 18:0 substrates, as determined with ratios of monoenes under saturated FA, and particularly on the n-7 ratio. The lipid profile was then monitored by TLC (Fig. 2b). Measurement of the density of bands confirmed the decrease in triglycerides for rats fed with the Scia diet (−36%). Furthermore, the FA profile showed that Scia was less prominent in the triglyceride fraction than in the phospholipid fraction (2.6% versus 7.1%). When considering triglycerides, the Scia diet reduced the monoene proportion from 31.7% to 19.5% of the total FA and affected each molecular species (Fig. 2c). As a consequence, the Δ9-desaturase indexes were impacted especially for the n-7 FA, as 16:0 substrate was slightly increased with the application of the Scia diet (from 25,2% to 29,9% of the total FA, data not shown). Further analyses showed that the triglyceride content in plasma was positively correlated to the n-7 and n-9 FA ratios (Fig. 2d). In addition, the concentration of the triglycerides in plasma was negatively correlated to the proportion of Scia contained in these lipids and a difference was markedly observable depending on the diet for each correlation.

### Effect of the Scia diet on plasma fractions

**Figure 3:**
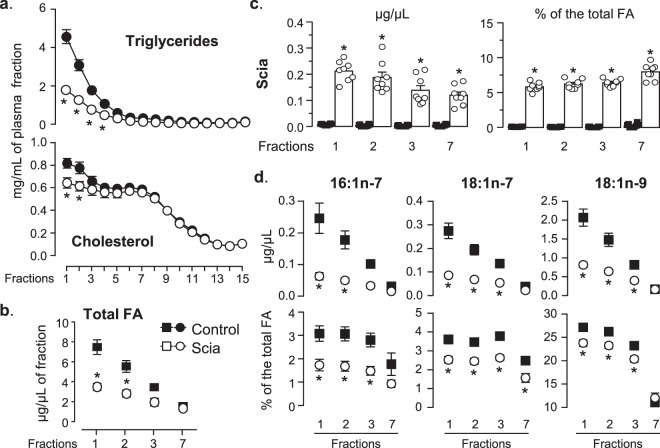
The effect of Scia on the lipid profile in the separated plasma fractions. Samples of blood plasma were ultra-centrifuged under an iodixanol gradient to separate out lipoproteins. (**a**) Cholesterol and triglycerides were enzymatically quantified in the fractions. (**b**) The FA content was estimated in the first three fractions and compared to the final fraction 7. (**c**) The Scia content in separate fractions was determined by GC. Results are expressed either as µg of FA per µL of fraction or as the % of the total FA. (**d**) Monoenes were quantified by GC analysis. Results are expressed either as µg of FA per µL of fraction or as the % of the total FA excluding Scia. The effect of diet and separated fractions was determined by ANOVA followed by a multiple comparisons test (*p < 0.05).

In parallel, the plasma samples were ultra-centrifuged under an iodixanol gradient to separate out the lipoproteins. Cholesterol and triglycerides were quantified in each fraction (Fig. 3). The Scia diet induced a reduction of cholesterol particularly in the first two fractions (−21%) (Fig. 3a). The Scia diet also greatly reduced triglycerides in the first four fractions by 60% to 54% when compared to the control diet. The FA profile of the first three fractions was further analyzed by GC and the result compared to that for fraction 7. Results showed that the Scia diet led to a reduction of the total FA content by half in the first fractions reflecting a similar pattern to that observed for triglycerides (Fig. 3b). Furthermore, when the FA profile was determined, the Scia content fell slightly as the fraction number increased, from 0.21 µg/µL to 0.11 µg/µL in samples relating to the Scia diet (Fig. 3c). It averaged 6% of the total FA in the first fractions, and up to 8% in the 7^th^ one. Other Δ5-olefinic acids present in very small amounts in the diet were also recovered from the plasma fractions (Table 2). 18:3Δ5,9,12 and its elongation product, 20:3Δ7,11,14, were both found in the plasma fractions, up to 26 ng/µL (0.7% of the total FA) and 5.5 ng/µL (0.2% of the total FA) respectively in the first fraction, and in decreasing concentrations in further fractions.

**Table 2:** The FA profile in fractions of blood plasma.

	Fraction 1		Fraction 2		Fraction 3		Fraction 7		F	D	I
	Control	Scia	Control	Scia	Control	Scia	Control	Scia			
10:0	0.0 ± 0.0	0.0 ± 0.0	0.0 ± 0.0	0.0 ± 0.0	0.0 ± 0.0	0.0 ± 0.0	0.0 ± 0.0	0.0 ± 0.0			
12:0	0.4 ± 0.4	0.0 ± 0.0*	0.3 ± 0.3	0.0 ± 0.0*	0.3 ± 0.3	0.0 ± 0.0*	0.1 ± 0.1	0.0 ± 0.0	*	*	*
14:0	0.7 ± 0.3	0.4 ± 0.1	0.7 ± 0.2	0.4 ± 0.2*	0.6 ± 0.2	0.4 ± 0.2	0.5 ± 0.3	0.5 ± 0.4		*	
16:0	19.2 ± 1.4	16.7 ± 2.4*	19.6 ± 1.7	16.2 ± 1.8*	19.1 ± 1.5	16.3 ± 1.8*	16.8 ± 1.8	17.5 ± 2.3		*	*
18:0	4.7 ± 0.5	6.3 ± 1.4*	5.1 ± 0.7	6.3 ± 1.3	6.0 ± 0.5	7.3 ± 1.3	11.3 ± 1.1	12.7 ± 1.8	*	*	
**Σ saturates**	**25.0** ± **0.5**	**23.4** ± **0.9**	**25.8** ± **0.6**	**23.0** ± **0.8**	**26.0** ± **0.5**	**24.0** ± **0.8**	**28.6** ± **0.7**	**30.8** ± **1.0**	*		
16:1 n-7	3.1 ± 0.9	1.8 ± 0.7*	3.1 ± 0.8	1.7 ± 0.5*	2.8 ± 0.8	1.5 ± 0.5*	1.8 ± 1.3	0.9 ± 0.5	*	*	
18:1 n-7	3.6 ± 0.7	2.5 ± 0.5*	3.5 ± 0.6	2.5 ± 0.4*	3.8 ± 0.6	2.7 ± 0.4*	2.5 ± 0.4	1.4 ± 0.8*	*	*	
20:1 n-7	0.3 ± 0.1	0.5 ± 0.2	0.2 ± 0.1	0.5 ± 0.1*	0.1 ± 0.2	0.4 ± 0.1*	0.0 ± 0.0	0.0 ± 0.1	*	*	
**Σ n-7**	**7.0** ± **0.4**	**4.8** ± 0.2*	**6.7** ± **0.4**	**4.7** ± 0.2*	**6.8** ± **0.3**	**4.6** ± 0.2*	**4.3** ± **0.7**	**2.3** ± 0.3*	*	*	
16:1 n-9	0.3 ± 0.1	0.5 ± 0.1	0.3 ± 0.0	0.5 ± 0.1	0.4 ± 0.3	0.4 ± 0.1	0.3 ± 0.3	0.2 ± 0.2			
18:1 n-9	27.2 ± 1.1	23.9 ± 2.0*	26.3 ± 1.3	23.3 ± 1.9*	23.3 ± 1.4	20.4 ± 1.9*	11.2 ± 1.1	10.9 ± 2.6	*	*	*
20:1 n-9	0.4 ± 0.0	0.6 ± 0.3	0.3 ± 0.1	0.7 ± 0.1*	0.3 ± 0.2	0.6 ± 0.1*	0.0 ± 0.0	0.1 ± 0.2	*	*	
20:3n-9	0.0 ± 0.0	0.0 ± 0.0	0.0 ± 0.0	0.0 ± 0.0	0.0 ± 0.0	0.0 ± 0.0	0.0 ± 0.0	0.0 ± 0.0			
**Σ n-9**	**27.9** ± **0.5**	**25.0** ± 1.0*	**26.9** ± **0.6**	**24.5** ± 0.9*	**24.0** ± **0.6**	**21.5** ± 0.9*	**11.4** ± **0.5**	**11.2** ± **1.2**	*	*	*
16:2n-6	0.0 ± 0.0	0.1 ± 0.1*	0.0 ± 0.0	0.1 ± 0.1*	0.0 ± 0.0	0.0 ± 0.1	0.0 ± 0.0	0.0 ± 0.1		*	
18:2n-6	25.6 ± 1.4	27.7 ± 2.8	25.2 ± 1.8	27.3 ± 2.6	24.7 ± 1.8	26.2 ± 2.6	19.8 ± 2.0	17.7 ± 2.1	*	*	
18:3 n-6	0.1 ± 0.1	0.1 ± 0.1	0.1 ± 0.1	0.0 ± 0.1	0.1 ± 0.1	0.0 ± 0.1	0.0 ± 0.0	0.0 ± 0.0	*	*	
20:2 n-6	0.4 ± 0.0	1.2 ± 0.2*	0.4 ± 0.2	1.3 ± 0.1*	0.4 ± 0.1	1.2 ± 0.1*	0.1 ± 0.2	0.6 ± 0.5*	*	*	
20:3 n-6	0.5 ± 0.1	0.4 ± 0.1	0.4 ± 0.1	0.4 ± 0.1	0.5 ± 0.1	0.4 ± 0.1	0.7 ± 0.3	0.2 ± 0.2*		*	*
20:4 n-6	5.7 ± 1.0	8.4 ± 2.1	6.4 ± 1.2	9.7 ± 2.9	9.5 ± 1.5	13.6 ± 2.9*	24.8 ± 4.7	29.8 ± 4.3	*	*	
22:4 n-6	0.4 ± 0.1	0.5 ± 0.2	0.4 ± 0.0	0.5 ± 0.2	0.4 ± 0.0	0.4 ± 0.2	0.2 ± 0.2	0.1 ± 0.1	*		
22:5 n-6	0.2 ± 0.0	0.1 ± 0.1	0.2 ± 0.0	0.1 ± 0.1	0.1 ± 0.1	0.0 ± 0.1	0.1 ± 0.1	0.0 ± 0.1		*	
**Σ n-6**	**32.9** ± **0.5**	**38.6** ± 1.1*	**33.2** ± **0.7**	**39.5** ± 1.2*	**35.8** ± **0.7**	**41.9** ± 1.2*	**45.7** ± **1.7**	**48.4** ± **1.5**	*	*	
18:3 n-3	3.1 ± 0.2	2.7 ± 0.6	3.0 ± 0.3	2.6 ± 0.3	2.6 ± 0.3	2.2 ± 0.3	1.0 ± 0.4	0.7 ± 0.3	*	*	
20:3 n-3	0.1 ± 0.1	0.5 ± 0.9	0.3 ± 0.6	0.4 ± 0.2	0.2 ± 0.1	0.3 ± 0.2	1.0 ± 1.7	0.3 ± 0.5			
20:5 n-3	0.4 ± 0.1	0.3 ± 0.2	0.4 ± 0.2	0.6 ± 0.1	0.6 ± 0.3	0.3 ± 0.1	1.4 ± 1.6	0.1 ± 0.1*		*	*
22:5 n-3	0.6 ± 0.1	0.6 ± 0.1	0.6 ± 0.1	0.6 ± 0.1	0.6 ± 0.1	0.5 ± 0.1	0.5 ± 0.1	0.4 ± 0.2	*		
22:6 n-3	2.3 ± 0.7	2.2 ± 0.5	2.2 ± 0.4	2.4 ± 0.5	2.4 ± 0.3	2.6 ± 0.5	3.9 ± 0.8	4.0 ± 0.7	*		
**Σ n-3**	**6.5** ± **0.2**	**6.2** ± **0.3**	**6.5** ± **0.2**	**6.5** ± **0.2**	**6.4** ± **0.1**	**5.9** ± **0.2**	**7.8** ± **0.7**	**5.6** ± 0.2*			
18:2Δ5,9	0.0 ± 0.0	0.0 ± 0.0	0.0 ± 0.0	0.0 ± 0.0	0.0 ± 0.0	0.0 ± 0.0	0.0 ± 0.0	0.0 ± 0.0			
18:3Δ5,9,12	0.0 ± 0.0	0.7 ± 0.5*	0.0 ± 0.0	0.6 ± 0.1*	0.0 ± 0.0	0.5 ± 0.1	0.0 ± 0.0	0.4 ± 0.3*		*	
20:2Δ5,11	0.0 ± 0.0	0.0 ± 0.0	0.0 ± 0.0	0.0 ± 0.0	0.0 ± 0.0	0.0 ± 0.0	0.0 ± 0.0	0.0 ± 0.0		*	
20:3Δ7,11,14	0.0 ± 0.0	0.2 ± 0.7*	0.0 ± 0.1	0.2 ± 0.6*	0.0 ± 0.1	0.1 ± 0.6	0.0 ± 0.3	0.0 ± 1.2	*	*	*
**Σ Δ5-olefinic**	**0.0** ± **0.0**	**0.9** ± 0.2*	**0.0** ± **0.0**	**0.8** ± 0.0*	**0.0** ± **0.0**	**0.6** ± 0.0*	**0.0** ± **0.0**	**0.4** ± 0.1*		*	
Others	0.7 ± 0.0	1.1 ± 0.0	0.8 ± 0.0	1.0 ± 0.0	1.0 ± 0.0	1.5 ± 0.0	2.2 ± 0.0	1.2 ± 0.0	*		

Plasma was ultra-centrifuged under iodixanol gradient to separate out the lipoproteins. Lipids were extracted from the fractions 1, 2, 3 and 7 by Folch’s method. The FA profile was determined by GC. Results are expressed in mass % of the total FA but excluding Scia. The statistical significance of the effect of diet and fractions was determined by ANOVA followed by a multiple comparisons test (*p < 0.05). F: Fraction, D: Diet, I: Interaction (Fraction x Diet).

Interestingly the proportions of monoenes also declined in the plasma fractions from rats fed the Scia diet (Fig. 3d). The strongest effect of the Scia diet was observed with respect to 16:1n-7 with a reduction of 45% compared to the control, whereas 18:1n-7 was reduced by 30% and 18:1-n9 by 12% in the first three fractions, representing an averaged decrease of 5.5% of the total FA. It should be noted that the animals had not been fasted prior to the trial. Thereby chylomicrons and VLDL were presumed present in the first fraction, while VLDL were also present up to the fourth fraction. Chylomicrons reflecting the diets did not impact on the results as the proportions of FA ranged from C16 to C18 in the fractions evolved whereas they were the same in both diets.

Finally, as Scia was previously known to cause an increase in 18:2n-6 after partial β-oxidation in the liver, its level in plasma fractions was checked. The Scia diet had the effect of inducing a global increase in n-6 FA without significantly increasing the proportion of 18:2n-6 (Table 2). On the contrary, one of the precursors of monoenes, palmitic acid or 16:0, was significantly reduced in the first fractions and represents the FA, together with 20:2n-6, that was particularly affected by the Scia diet. Considering that plasma triglycerides are partly *de novo* synthesized in the liver and then secreted through VLDL, which are notably present in the first two fractions, further attention was directed at the role of Scia on the liver.

### Effect of the Scia diet in the liver

**Figure 4:**
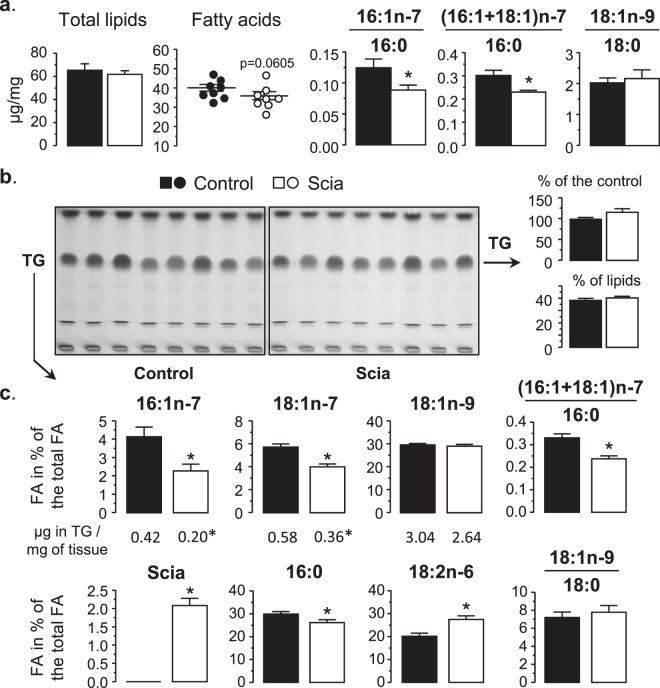
The effect of Scia on the lipid profile of the liver. Lipids in the liver were extracted by Folch’s method and the FA profile was determined by GC. Lipid classes were further separated by TLC. (**a**) Lipid content, FA concentration and Δ9-desaturase indexes were determined on total lipids. (**b**) The TLC profiles were then performed depending on the diets (2.5 mg of liver sample per well). Triglycerides were estimated by relative quantification of the density of bands. (**c**) FA proportions and Δ9-desaturase indexes were determined on the triglyceride fractions. Results on FA from TG are expressed as a % of total FA including Scia. The significance of the difference between diets was evaluated by a *t*-test (*p < 0.05).

A first observation was that the Scia diet did not affect significantly the lipid content of liver samples, although the FA content tended to decrease (Fig. 4a). It did though partially affect the Δ9-desaturase indexes, in reducing the n-7 FA ratios without impacting on the ratio of 18:1n-9 to 18:0. When the lipid profile was revealed by TLC, no significant difference could be discerned between the two diets (Fig. 4b). In particular, the triglyceride species was not changed by the Scia diet. Nevertheless, Scia was detected in the liver reaching 0.20 µg in triglycerides per mg of tissue. Furthermore, an increase in 18:2n-6, subsequent to the incorporation of Scia in cells, was also observed (Fig. 4c). The most interesting result concerned the Δ9-desaturase indexes measured in triglycerides. Indeed both 16:1n-7 and 18:1n-7 were significantly reduced as part of the triglycerides by the Scia diet, leading to the reduced n-7 FA ratios, whereas the presence of 16:0 was significantly decreased as well. On the other hand, neither the fatty acid 18:1n-9 nor the associated Δ9-desaturase index were affected. As hepatic triglycerides may be native from neo-remodeling of lipids in the liver or from blood after cell incorporation, analyses were further focused on lipid metabolism with specific attention to the transcription factors and the enzymes involved in the FA synthesis.

**Figure 5:**
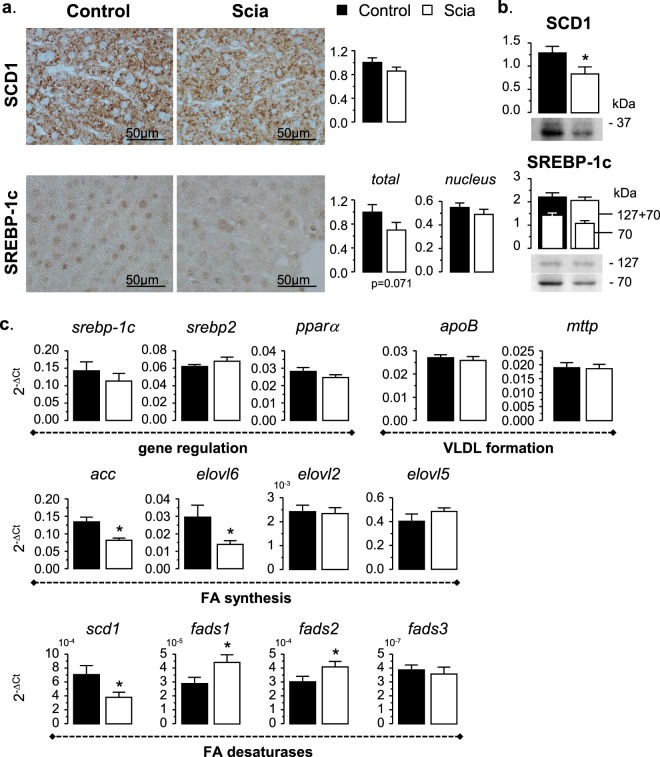
The effect of Scia on the expression of modulators involved in the lipid metabolism of the liver. (**a**) The protein expression and location of SCD1 and SREBP-1c was determined on liver sections by immunohistology. The density of the brown staining was quantified and expressed in arbitrary units. (**b**) The protein expression was further estimated by western-blot after normalization with GAPDH and expressed in arbitrary unit. The averaged effect of the Scia diet was illustrated by the blot of a pooled sample from the 8 animals. (**c**) Gene expression of s*cd1* and of actors involved in the regulation of lipid metabolism was estimated by real time qPCR: *srebp-1c* (sterol regulatory element binding protein-1c) and *srebp2* are involved in the *de novo* synthesis of FA and cholesterol respectively, *pparα* (peroxisome proliferator-activated receptor α) is implicated in the FA oxidation, *acc* (acetyl-CoA carboxylase) catalyzes the formation of malonyl-CoA implied in the *de novo* synthesis of saturated FA, *elovl6* (elongation of very long-chain fatty acid 6) participates to the elongation of 16:0 into 18:0, *elovl2* is involved in the elongation of C20 and C22 PUFA, *elovl5* elongates C18 PUFA, *elovl5,6* also promotes the elongation of 16:1n-7 into 18:1n-7, *scd1* (stearoyl-CoA desaturase 1) introduces a double bond in Δ9 of saturated FA, *Fads1* (fatty acid desaturase 1) and *Fads2* desaturate in the positions Δ5 and Δ6 of FA respectively, *Fads3* was recently shown as a Δ14Z sphingoid base desaturase^50^, *apoB* (apolipoprotein B) is part of the VLDL particles and *mttp* (microsomal triglyceride transfer protein) participates to the triglyceride packing into VLDL. The significance of the difference between diets was evaluated by a *t*-test (*p < 0.05).

Liver sections were immunostained with the hepatic Δ9-desaturase SCD1, and SREBP-1c known to upregulate lipogenic enzymes such as SCD1 (Fig. 5a). Results showed that tissue samples taken from rats fed the Scia diet displayed a decrease in the staining of SCD1 by 15% (p = 0.0928), when compared to the control. The Scia diet did not affect the immunostaining of the cell nucleus with respect to SREBP-1c, whereas a decrease in the total quantity (−30%) was observed (p = 0.0712), suggesting a possible effect on protein activation through its subcellular location. When performing western-blots on liver extracts, protein expression of SCD1 was reduced by 35% in the case of samples relating to the Scia diet (Fig. 5b). In contrast, no significant effect was observed on SREBP-1c, regardless of the protein isoform. This effect was confirmed by the gene expression estimated by quantitative PCR (Fig. 5c). The Scia diet inhibited the gene expression of *scd1* by 45%, without impacting on the second isoform *scd2* weakly expressed in the liver (not shown).

With respect to the precursors to monoene synthesis, the Scia diet also reduced the gene expression of acetyl-CoA carboxylase (ACC, −39%), which catalyzes one of the first steps in the synthesis of saturated FA. The expression of elongase *elovl6*, which is implied in the elongation of 16:0 into 18:0, was also greatly reduced (−53%). On the contrary, there was no effect on expression of the other isoforms *elovl2* and *elovl5* mainly involved in elongation of n-6 and n-3 polyunsaturated FA (PUFA). Regarding the metabolism of polyunsaturated FA, the Scia diet increased the expression of *Fads1* (+54%) and *Fads2* (+36%), encoding respectively for the Δ5- and Δ6-desaturases, but had no impact on *Fads3*. Yet, this gene regulation did not influence the FA profile in liver as the Scia diet only induced a significant increase of 18:2n-6, that was then elongated into 20:2n-6.

*De novo* lipogenesis is controlled by transcription factors such as *srebp-1c* or *srebp2*, which are involved in FA and cholesterol synthesis respectively, whereas lipid oxidation is regulated by peroxisome proliferator-activated factor (ppar) α. Surprisingly the results produced from this study showed that the Scia diet did not impact on the gene expression of either *pparα* or *srebp-1c* or *srebp2* as previously observed in *in vitro* studies^17^. Neither did the Scia diet modify the gene expression of apolipoprotein B (*apoB*), produced during the nascent VLDL formation, nor of microsomal triglyceride transfer protein (*mttp*), implied in triglyceride packing. Overall these results showed that Scia, independently of its own metabolism, modulated the synthesis of mono- and unsaturated FA in the liver of non-fasting rats mainly by inhibiting the activity and expression of SCD1.

### Effect of the Scia diet on oxidized derivatives of fatty acids

**Figure 6:**
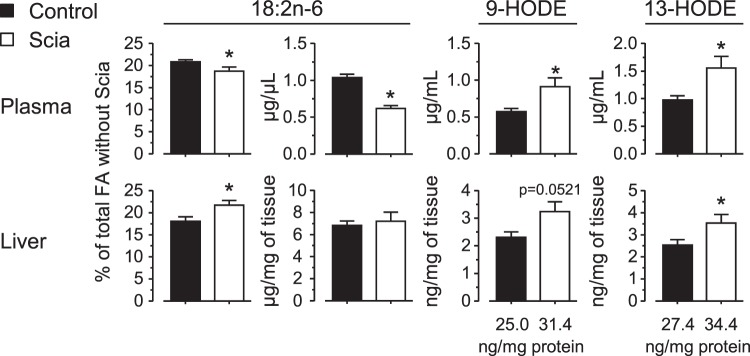
The effect of Scia on the oxidized derivatives of linoleic acid. 9-HODE and 13-HODE, the main oxidized metabolites of 18:2n-6, were quantified in plasma and liver by LC-QQQ. Results were set alongside the quantitation of linoleic acid. The significance of the difference between diets was estimated by a *t*-test (*p < 0.05).

Considering that the Scia diet modulated the FA metabolism, the inflammatory status was further evaluated by the measurement of derivatives of FA such as oxidized linoleic acid metabolites (OXLAM), eicosanoids and docosanoids in liver and plasma. The analysis was first focused on derivatives of 18:2n-6 (Fig. 6). The Scia diet induced a significant increase in 9-hydroxyoctadecadienoic acid (9-HODE) and 13-HODE in both tissues. This result was consistent with the proportion of 18:2n-6 in liver samples. On the contrary, neither eicosanoids derived from 20:4n-6 and 20:5n-3, nor docosanoids from 22:6n-3 were significantly affected by the Scia diet (Supplemental Table 1).

## Discussion

Previous *in vitro* studies have shown that the presence of Scia could lower the formation and secretion of triglycerides in cultured hepatocytes^17^. This effect was in part supported by the inhibition of the expression and the activity of SCD1. In the present *in vivo* study, the molecular mechanism of the action of Scia ingested by rats was investigated. It emerged that Scia reduced the gene and protein expression of SCD1 in the liver. This effect was confirmed by the reduced proportion of 16:1n-7 and 18:1n-7, but no change was observed in the level of 18:1n-9, considered as the main product of SCD1 involved in the *de novo* triglyceride synthesis^26^. These results contrast with experiments with rats performed by Endo *et al*., who compared diets containing corn, soybean and *Torreya nucifera* oils^15^. These authors reported a large fall in the triglyceride concentration in the liver when rats consumed the *Torreya* oil but without investigating the effect on monoenes from hepatic triglycerides. Endo *et al*. studied thereafter the nutritional effect of Scia purified from *Torreya nucifera* but the FA was supplied as the ethyl ester in the diet^16^. After a two-week trial, they observed a 50% reduction of the liver triglyceride concentration in rats previously fasted prior to the tissue collection. This effect was associated with a reduction of monoenes, which might lead to the idea that it was due to the increase in Scia, which is absent from the control diet. However, the level of monoenes synthesized by SCD1 is highly correlated to the synthesis and secretion of triglycerides in hepatocytes, as reported by Miyazaki *et al*.^27^ and confirmed in previous work performed on rat cultured hepatocytes^17^. The apparent discrepancy between these studies may be explained by the design of the experiment, which used fasted rats in one case and non-fasted rats in the other. Hepatic FA came either from the *de novo* biosynthesis or from the diet itself. If n-7 monoenes were negligible from the diet, they must have arisen from the endogenous FA metabolism. When detected in the liver, the effect of Scia was significant on both 16:1n-7 and 18:1n-7 suggesting an effect on SCD1. In contrast, 18:1n-9 was partly biosynthesized and also originated from the diet in which it made up 30% of the fatty acids. The Scia diet did not lower the level of 18:1n-9 in the liver but the expected decrease of its biosynthesis could have been hidden by the 18:1n-9 supply by the diet. A similar explanation may be offered for triglycerides as no influence from the diet was observed. The liver was already rich in triglycerides from remnant chylomicrons and it further produced and released triglycerides with neosynthesized FA. Two different pools of triglycerides thus coexisted in the liver but no distinction was possible in this study. Hence the metabolic impact of Scia was negated by the postprandial conditions, considering that tissues were collected from non-fasted animals in the morning. This argument is supported by Lounis *et al*.^28^. They showed *in vitro* that 18:1n-9 treatment of HepG2 cells can restore the expression of SREBP-1 and increase *de novo* lipogenesis, even with SCD1-deficient hepatocytes. In the present study, the high dose of 18:1n-9 from the diet would negate the inhibitory effect of Scia on SCD1 expression and activity. At last 16:1n-7 and 18:1n-7 were the best indicators of this inhibition in the physiological conditions used in the present study.

The expression of SCD1 is known to be lowered by fish oils or n-6 and n-3 PUFA such as 18:2n-6, eicosapentaenoic acid (EPA, 20:5n-3) or docosahexaenoic acid (DHA, 22:6n-3)^29–32^. Previous related work also showed an inhibiting effect of several eicosatrienoic acids on *in vitro* SCD1 activity depending on their structure, with a predominant effect observed with 20:3n-6, followed by 20:3n-9 and Scia, but no effect from 20:3n-3^17^. Other authors also demonstrated that dietary 18:2n-6 mediated the downregulation of the SCD1 gene expression in mice^33,34^. In this study, the Scia diet induced an increased proportion of 18:2n-6 in the liver. Therefore, the effects of Scia could be explained by those of 18:2n-6 as this n-6 PUFA is described as a modulator of SCD1 acting by inhibiting the maturation of SREBP-1c. In this work, Scia reduced the expression of SCD1 in the liver but independently of the expression and sub-location of SREBP-1c. Overall these observations contrast with the anti-lipogenic action of dietary PUFA described as inhibitors of SREBP-1c, and resulting in the reduced triglyceride synthesis^35^ and plasma VLDL release^36,37^. This supports the idea of additional effects beyond those mediated by SREBP-1c, in parallel to the non-fasting conditions. The same previous work *in vitro* suggests that the enrichment of the endoplasmic reticulum membranes with Scia would modulate the activity *in situ*; precise mechanisms to account for this remain to be explored. Another line of approach could also imply oxidized derivatives of FA. Scia was described as a modulator of inflammation as it reduced the production of PGE_2_ from macrophages, infected epithelial cells or microglial BV-2 cells *in vitro*^10,11,13^. The present study did not strengthen this hypothesis. The Scia diet did not significantly change the level of eicosanoids although the measurement was performed in plasma and liver and could be further investigated in more relevant tissues for this purpose. Nevertheless, the Scia diet raised the plasma and liver content of 9/13-HODE. These OXLAMs, and maybe others non-detected in this study, may be part of the FA metabolism modulated by Scia. They display pleiotropic effects that are either beneficial or prejudicial, depending on the context. They are biomarkers of inflammatory responses or oxidative stress, indicators of intensive physical activity or of some cancers. 13-HODE is considered as a potent inducer of reactive oxygen species production in endothelial cells and may influence functions of endothelium as a mediator of oxidative stress^38^. 9-HODE and 13-HODE were also described as biomarkers of post-exercise inflammation since their concentrations increase following prolonged and intensive exercise^39^. They were incriminated in neuropathic pain, particularly concerning mechanical allodynia and thermal hyperalgesia^40^. In breast cancer, HODEs were the most important oxylipin species upregulated in plasma of cancer patients^41^ whereas they were lowered in serum of colorectal cancer patients^42^. HODEs were widely described for their damaging effects on health but contradictory results are emerging from the literature. 13-HODE was indeed described for anti-atherogenic properties through inhibition of adhesion of several blood cells to the endothelium^43^. Produced by endothelial cells, it would behave like an anti-inflammatory metabolite as a ligand for PPAR α and γ. Its synthesis led to reduce angiogenesis in colon and prostate cancer^44^ or enhanced the axonal outgrowth in primary cortical neurons during development of rats in a gender-dependent manner^45^. Altogether, these works underline the pleiotropic dimension of action of HODEs. In the present study, the endogenous formation of 9/13-HODE after a Scia enriched-diet could provide new metabolic answers. Further investigations would be of interest for that purpose.

After the synthesis in the liver, triglycerides are packed in VLDL and secreted in the blood. In this respect, Scia reduced lipid plasma mainly due to the lowered level of triglycerides from VLDL. The most important effect was observed in the first fraction of ultra-centrifuged plasma, which contained VLDL and chylomicrons as well. As food consumption was similar for the test and control diets, it can be concluded that the effect of Scia was mainly to reduce the lipid content of VLDL. Circulating triglycerides found in the plasma contained lower levels of monoenes, even the 18:1n-9, which is consistent with the preceding hypothesis relating to the Scia effect on SCD1 in the liver and considering the packaging of newly synthesized triglycerides in VLDL. Noting that the Scia diet reduced *de novo* lipogenesis in the liver by modulating the SCD1 activity, the consequences on the plasma triglycerides were underlined. Other lipogenic enzymes were thus checked in this study and data produced supported the idea of the inhibition of saturated FA synthesis as implied by *acc* and *elovl6* gene expression previously observed *in vitro*^17^. This result was expected as Endo *et al*. had previously showed a significant decrease in the activity of glucose-6-phosphate dehydrogenase with *Torreya nucifera* oil^46^, this enzyme providing the NAPDH necessary for the first steps of FA synthesis. This observation is consistent with another study performed using purified Scia, where the activity of malic enzyme and fatty acid synthase among others were all inhibited by the Scia-enriched diet^16^. In this case, the present study shows that the Scia had enhanced the gene expression of *Fads1* and *Fads2* in the liver. No increase in the concentration of long chain PUFA was observed. The Δ6 index (18:3n-6/ 18:2n-6) and Δ5 index (20:4n-6/ 20:3n-6) of desaturation for n-6 FA were slightly higher (data not shown) but presumably as a result of the 18:2n-6 increase produced with the Scia diet. Different studies suggest an inhibiting effect of long chain PUFA such as EPA, DHA or arachidonic acid (20:4n-6) on the Δ5- and Δ6-desaturase activity, particularly with respect to n-6 FA biosynthesis^47^. The 18:2n-6 and 18:3n-3 precursors were in fact revealed as activators of desaturases involved in the biosynthesis of longer chain PUFA^48,49^. In the present study, Scia, which has a similar structure to arachidonic acid, presents an ambivalent behavior being like a long chain PUFA acting as an inhibitor of SCD1, and being also like a precursor of PUFA acting as an activator of Δ5/Δ6-desaturases. Both actions on lipogenic enzymes were effectively to favour PUFA synthesis.

Finally, this study confirmed the benefit of Scia on lowering triglyceridemia when fed to rats that had not been previously fasted. The non-fasting state for triglyceride tests is more and more widespread for evaluating the risk of heart disease and the metabolic syndrome. In this study, rats were normotriglyceridemic and supplemented with a diet equilibrated in C16 and C18 FA, and which contained a significant supply of PUFA precursors (representing 7.6% and 1.2% of energy for the 18:2n-6 and 18:3n-3 fatty acids respectively). Scia, widely present in edible seeds, behaved like an inhibitor of the hepatic Δ9-desaturase SCD1. The inclusion of such food ingredients in the daily diet could promote human health by reducing metabolic disorders without resorting to medication.

## Supplementary information

Supplementary information
