# Supplementary material for: Sciadonic acid derived from pine nuts as a food component to reduce plasma triglycerides by inhibiting the rat hepatic Δ9-desaturase
Source: Sci Rep. 2020 Apr 10;10:6223. doi: 10.1038/s41598-020-63301-3 (PMC7148351; doi:10.1038/s41598-020-63301-3)
Supplement: Supplementary file 1 — Supplementary information [file 41598_2020_63301_MOESM1_ESM.pdf]

Sciadonic acid derived from pine nuts as a food component to reduce plasma triglycerides by inhibiting the rat hepatic Δ9-desaturase.

Frédérique Pédrono[1,2]*, Nathalie Boulier-Monthéan[1,2], Françoise Boissel[1,3], Jordane Ossemond[2], Roselyne Viel[4], Alain Fautrel[4], Justine Marchix[5], Didier Dupont[2]

[1] AGROCAMPUS OUEST, Rennes, France
[2] INRAE Science et Technologie du Lait et de l'Œuf, équipe Bioactivité et Nutrition, Rennes, France
[3] INRAE Science et Technologie du Lait et de l'Œuf, équipe Séchage, Matrices concentrées et Fonctionnalités, Rennes, France
[4] Université de Rennes1, Inserm, CNRS, Plateforme d'histopathologie H2P2, Biosit, Biogenouest, France
[5] Cincinnati Children's Hospital Medical Center, Division of Pediatric General and Thoracic Surgery, Cincinnati, United States
* Corresponding author: Institut Agro, AGROCAMPUS OUEST, 65 rue de Saint-Brieuc, 35042 Rennes cedex, France, frederique.pedrono@agrocampus-ouest.fr, phone number (33) 2 23 48 55 46, ORCID ID 0000-0002-4973-7738

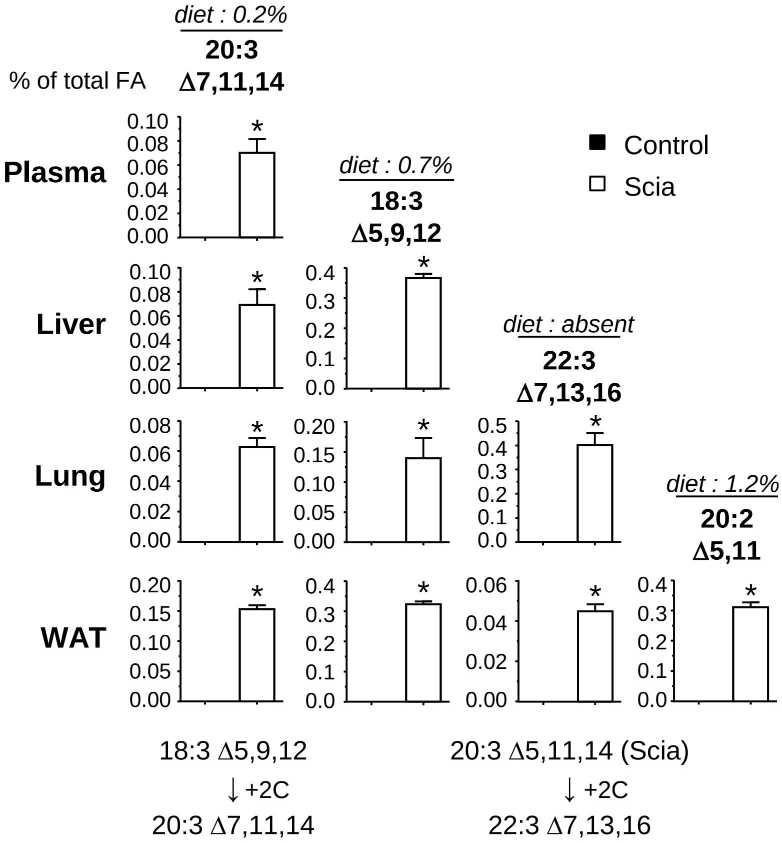

Supplemental Figure 1. Metabolism of Scia and the derivatives of Δ5-olefinic acids.
Lipids from tissue samples were extracted by Folch's method and the FA profile subsequently determined by GC. Results focused on Δ5-olefinic acids and their elongation products found with the Scia diet. Only tissues where the Δ5-olefinic acids differed with the Scia diet are represented. The significance of the difference between diets was estimated by a *t*-test (* $p<0.05$).

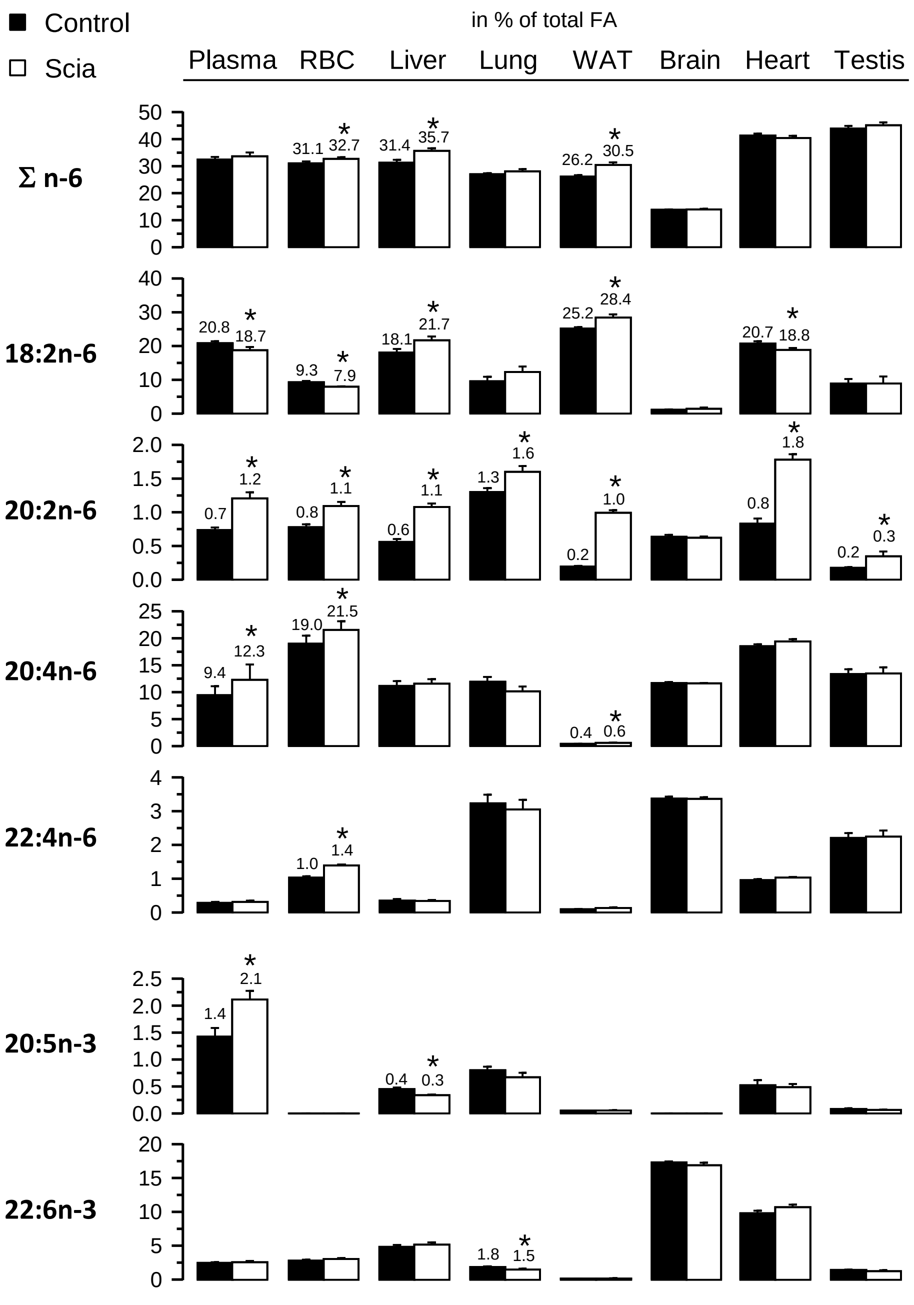

Supplemental Figure 2. Impact of Scia on the FA profile.

Tissue lipids were extracted by Folch's method and the FA profile was determined by GC. Results focused on PUFA, mainly n-6 and to a lesser extent n-3, that were specifically modulated with the Scia diet. The significance of the difference between diets was measured by a *t*-test (* $p<0.05$).

Supplemental Table 1. Effect of Scia on oxidized derivatives of FA.

The FA derivative profile was determined from plasma and liver by LC-QQQ.

Concerning FA, concentration ([a]) of 20:4n-6 is expressed in µg/µL of plasma or mg of liver, of 20:5n-3 in ng/µL or mg and of 22:6n-3 in ng/µL or µg/mg. Proportions of FA ([b]) are in % of the total FA without Scia.

Concerning derivatives, results were expressed in ng/mL of plasma and ng/mg of proteins for the liver. The statistical significance of the effect of diet and fractions was determined by ANOVA with a multiple comparisons test (* p<0.05). T : Tissue, D : Diet, I : Interaction (Tissue x Diet).

PG: Prostaglandin, TX: Thromboxane, LX: Lipoxin, (H)ETE: (Hydroxy)Eicosatetraenoic acid, HEPE: Hydroxyeicosapentaenoic acid, EET: Epoxyeicosatrienoic acid, HDoHE: Hydroxydocosahexaenoic acid, PDx: Protectin DX, Rv: Resolvin, MaR: Maresin.

		Plasma			Liver					
		Control		Scia	Control		Scia	T	D	I
**20:4n-6 derivatives**	20:4n-6a	0.5 ± 0.0		0.4 ± 0.0	4.2 ± 0.2		3.7 ± 0.2	*		
	20:4n-6[b]	9.4 ± 0.6	*	12.3 ± 1.0	11.2 ± 0.9		11.6 ± 0.8			
	PGF2α	3.0 ± 0.5		2.3 ± 0.4	3.3 ± 0.7		4.7 ± 1.0	*		
	PGE2	10.1 ± 1.3		8.1 ± 1.5	2.8 ± 0.7		2.9 ± 0.6	*		
	8-isoPGA2	5.1 ± 1.3		4.0 ± 0.5	0.5 ± 0.1		0.7 ± 0.2	*		
	PGD2	22.9 ± 4.6		20.9 ± 3.7	1.7 ± 0.6		1.3 ± 0.2	*		
	15-dPGJ2	0.3 ± 0.1		0.2 ± 0.0	0.0 ± 0.0		0.0 ± 0.0	*		
	TXB2	3.5 ± 0.5	#	2.4 ± 0.4	1.3 ± 0.6		1.2 ± 0.3	*		
	LXA4	103.1 ± 13.2		80.7 ± 12.7	0.7 ± 0.4		1.8 ± 0.7	*		
	LXB4	10.4 ± 0.9	#	8.4 ± 0.7	0.0 ± 0.0		0.0 ± 0.0	*		
	5-HETE	1908.7 ± 94.5		1709.9 ± 79.7	5.0 ± 1.0		7.1 ± 1.0	*		
	5-oxoETE	113.4 ± 11.4		109.1 ± 17.2	4.8 ± 1.2	#	7.3 ± 1.0	*		
	8-HETE	80.4 ± 4.8		88.0 ± 5.5	2.1 ± 1.2		1.0 ± 0.1	*		
	12-HETE	226.9 ± 18.7		208.0 ± 12.5	3.7 ± 1.1		2.4 ± 0.3	*		
	15-HETE	180.1 ± 11.7		187.4 ± 12.0	4.8 ± 0.7		6.3 ± 0.7	*		
	5,6-EET	2.8 ± 0.6		2.4 ± 0.5	1.0 ± 0.2		1.0 ± 0.2	*		
	5,6-DiHETE	0.5 ± 0.1		0.5 ± 0.0	0.0 ± 0.0		0.0 ± 0.0	*		
	8,9-EET	4.1 ± 0.7		3.2 ± 0.7	1.0 ± 0.2		0.9 ± 0.1	*		
	11,12-EET	1.7 ± 0.2		1.4 ± 0.2	0.6 ± 0.1		1.3 ± 0.7			
	14,15-EET	2.0 ± 0.2		1.7 ± 0.2	0.6 ± 0.2		0.6 ± 0.1	*		
**20:5n-3 derivatives**	20:5n-3[a]	70.2 ± 7.8		69.1 ± 6.2	171.1 ± 9.4	*	109.7 ± 3.7	*	*	*
	20:5n-3[b]	1.4 ± 0.2	*	2.1 ± 0.2	0.5 ± 0.0		0.3 ± 0.0	*	*	*
	PGE3	0.3 ± 0.1		0.3 ± 0.1	0.3 ± 0.1		0.3 ± 0.1	*		
	18-HEPE	17.6 ± 2.0		17.5 ± 1.6	0.7 ± 0.2		0.7 ± 0.1	*		
**22:6n-3 derivatives**	22:6n-3[a]	123.4 ± 5.1		84.1 ± 6.6	1.8 ± 0.1		1.6 ± 0.1	*		
	22:6n-3[c]	2.5 ± 0.1		2.6 ± 0.2	4.8 ± 0.3		5.1 ± 0.4	*		
	14-HDoHE	181.8 ± 20.9		192.0 ± 18.8	7.4 ± 5.3		2.2 ± 0.2	*		
	17-HDoHE	218.5 ± 25.1		249.0 ± 25.6	2.0 ± 0.5		1.8 ± 0.2	*		
	PDx	11.1 ± 1.2		11.4 ± 1.6	0.0 ± 0.0		0.0 ± 0.0	*		
	RVD1	0.8 ± 0.1		0.7 ± 0.1	0.0 ± 0.0		0.0 ± 0.0	*		
	RvD2	3.2 ± 0.2		2.6 ± 0.5	0.0 ± 0.0		0.0 ± 0.0	*		
	RvD5	8.0 ± 1.0		7.7 ± 1.0	0.0 ± 0.0		0.0 ± 0.0	*		
	7MaR1	21.7 ± 1.8		21.4 ± 2.4	0.0 ± 0.0		0.0 ± 0.0	*		

# p=0.06
